# The Impact of an 8-Week Supplementation with Fermented and Non-Fermented Aronia Berry Pulp on Cardiovascular Risk Factors in Individuals with Type 2 Diabetes

**DOI:** 10.3390/nu15245094

**Published:** 2023-12-13

**Authors:** Christine B. Christiansen, Per B. Jeppesen, Kjeld Hermansen, Søren Gregersen

**Affiliations:** 1 Department of Clinical Medicine, Aarhus University, Palle Juul-Jensens Boulevard 11, 8200 Aarhus N, Denmark; per.bendix.jeppesen@clin.au.dk (P.B.J.); kjeld.hermansen@clin.au.dk (K.H.); soeren.gregersen@aarhus.rm.dk (S.G.); 2 Department of Endocrinology and Internal Medicine, Aarhus University Hospital, Palle Juul-Jensens Boulevard 99, 8200 Aarhus N, Denmark; 3 Steno Diabetes Center Aarhus, Palle Juul-Jensens Boulevard 11, 8200 Aarhus N, Denmark

**Keywords:** *Aronia melanocarpa*, type 2 diabetes, dietary supplement, antioxidants, polyphenols, anthocyanins, randomized controlled trial

## Abstract

Aronia berries contain antioxidants that may be health-promoting, e.g., demonstrated positive effects on hypertension and dyslipidaemia. There is a close link between cardiovascular diseases and hypertension and dyslipidaemia, and cardiovascular events are the leading cause of death among subjects with type 2 diabetes (T2D). Thus, we investigated the effect of an 8-week supplementation with fermented aronia extract (FAE), non-fermented aronia extract (AE), and placebo on cardiovascular risk factors. Snack bars were produced containing 34 g (37%) aronia extract, or 17 g (21%) wheat bran for placebo, as well as raisins and coconut oil. The study was randomized and blinded with a triple-crossover design. We examined the effects of aronia extracts on blood pressure, adiponectin, and high-sensitive C-reactive protein, and found no effects. After supplementation with placebo, there were significantly higher blood concentrations of total cholesterol, LDL-cholesterol, and HDL-cholesterol, with the placebo group showing significantly higher increases in total cholesterol and LDL-cholesterol than the AE group. Furthermore, we observed an increase in HDL-cholesterol in the FAE group and an increase in triglyceride in the AE group. Thus, we assume that the raisins may have increased the participants’ cholesterol levels, with both AE and FAE having the potential to prevent this increase.

## 1. Introduction

The aim of this study was to examine the impacts of aronia extract, fermented aronia extract, and placebo on cardiovascular risk parameters in subjects with type 2 diabetes (T2D). T2D represents a major health burden [1,2], driven in particular by the high occurrence of cardiovascular events associated with it [3]. Around 65% of individuals with T2D die from a cardiovascular event, highlighting the need for improved treatment [3]. It has been established that there is a close link between the occurrence of cardiovascular events and the presence of dyslipidaemia and hypertension in T2D [3]. 

*Aronia melanocarpa* is a small shrub that produces small, nearly black berries [4]. The berry has a very high content of antioxidants, which has led to the exploration of their clinical application possibilities [5,6,7]. The primary antioxidants in aronia are anthocyanins, proanthocyanidins, and phenolic acids [4,5]. Despite conflicting evidence, several in vitro studies, in vivo animal studies, and human studies have shown positive outcomes on blood glucose and lipid levels after a supplementation with aronia [8]. Against dyslipidaemia, which involves increased triglyceride and LDL-cholesterol and decreased HDL-cholesterol, statins effectively decrease LDL-cholesterol and improves triglyceride and HDL-cholesterol levels to some degree [9]. One of the most common side effects is myalgia, which is highly likely to be the main reason that many individuals choose to discontinue statin treatment [10,11]. In contrast to statins, antihypertensive agents are better tolerated with higher compliance [12,13,14]. Nevertheless, a study by Naruszewicz et al. reported decreases in systolic and diastolic blood pressure of 11 and 7 mmHg, respectively, in patients with previous myocardial infarction after a supplementation with aronia for six weeks, even though half of the participants received ACE inhibitors [15]. Aronia supplementation may also have positive effects in subjects with previous myocardial infarction and have implications for blood pressure improvements. Aronia berries have a high content of polyphenols that may explain the anti-inflammatory and antioxidant properties [16,17]. Since hypertension is associated with oxidative stress and low-grade inflammation, a supplementation with aronia may have a positive impact on blood pressure [17]. Thus, aronia supplementation may be a natural and well-tolerated, cost-effective alternative to statin treatment and adjunct to antihypertensive agents. 

However, in comparison to many other polyphenols, those specific to aronia berries have low bioavailability and limited absorption from the gastrointestinal tract [18]. For some compounds, less than 1% is absorbed and detected in the blood after consumption [18,19,20]. The low bioavailability may reduce its usability as an antioxidant supplement. However, the bioavailability of aronia may be improved after fermentation, since the process reduces the compounds to simpler structures with higher bioavailability [21,22]. Most human studies that examine the effects of aronia on various diseases involve non-blinded investigations without a control group. In the present study, we looked at the effects on cardiovascular risk factors of an eight-week supplementation with aronia, both fermented and non-fermented, in individuals with T2D. 

We hypothesized that fermented aronia extract (FAE) improves glycaemia and lipidaemia in T2D more efficiently than both non-fermented aronia extract (AE) and placebo. The aim of the present article is to report secondary outcomes regarding the impact of FAE and AE on blood pressure, blood lipids, adiponectin, and high-sensitive C-reactive protein (hs-CRP). Our results on glycaemia have been published previously [23]. 

## 2. Materials and Methods

### 2.1. Test Products

The study design and test product composition have been described in detail previously [23]. All test products were provided as snack bars twice daily and contained raisins (55 g per day) and coconut oil (3 g per day). In addition, the test products contained either fermented aronia extract or non-fermented aronia extract (34 g), and the placebo contained wheat bran (17 g) together with water (5 g) and colorants. Aronia extracts were made from berries that had been harvested in September 2020 and stored frozen. Snack bars were produced throughout the study to ensure durability and high quality. Each time a new batch of snack bars were produced, the aronia berries were slowly thawed and cold-pressed. For the fermentation, aronia pulp was blended with a bacterial culture and allowed to ferment in a plastic bag to maintain humidity during the process. A singular analysis of the bacteria composition in the fermented aronia confirmed an upregulation of the added bacteria. Then, the pulp was either used unfermented directly in the snack bars or was fermented before being used in the snack bars. The dry matter percentage in the pulp was around 40%. Lastly, the aronia extracts, e.g., aronia pulp and fermented aronia pulp, were blended with the remaining ingredients. All snack bars including placebo was kindly provided by Elkaerholm (Egtved, Denmark). 

The group receiving fermented aronia extract will be referred to as FAE and the group receiving non-fermented aronia extract as AE. The aronia extracts were based on aronia berry pulp. The FAE and AE groups were supplemented with snack bars that administered approximately 893 mg or 533 mg anthocyanins, respectively, per day. Measurements on anthocyanin content were made by Elkaerholm (Egtved, Denmark) prior to the study, and since anthocyanins decay over time, the amount of provided anthocyanins might have decreased towards the end of the study. In order to minimize the decay, the supplements were stored in the dark at −18 °C. The nutritional composition is outlined in Table 1.

### 2.2. Study Design and Participants

This study was carried out at the Department of Endocrinology and Internal Medicine, Aarhus University Hospital, Denmark, spanning the period from December 2020 to April 2022. The study adhered to the principles outlined in the Declaration of Helsinki and received approval from The Central Denmark Region Committees on Health Research Ethics (Journal no. 1-10-72-102-19). Furthermore, the study was enlisted on ClinicalTrials.gov (NCT04647175).

**Figure 1:**
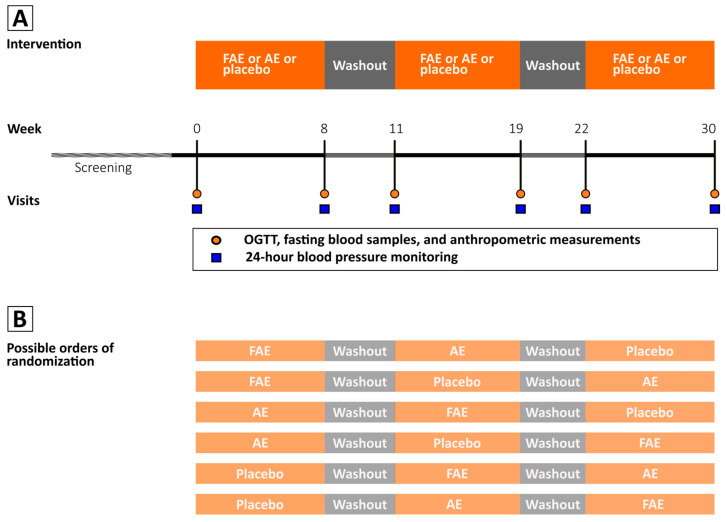
Study design. (**A**) Each intervention had a duration of eight weeks. Intervention periods were separated by washout periods with a duration of three weeks, resulting in a total study duration of 30 weeks. Before and after each intervention period, 24 h blood pressure and fasting blood samples were taken, which is marked with orange dots. (**B**) The order of the interventions was random with six possible orders.

Advertisements in local newspapers, a dedicated recruitment website (“forsoegsperson.dk”), and various social media platforms were used to get in contact with possible study candidates. Thereafter, the candidates were given all study information orally and in writing, along with at least one week for consideration. Interested candidates were screened for eligibility, and if they fulfilled the study criteria, a written consent was obtained. This consent could be withdrawn at any point in the study. Participants could be included if they had a diagnosis of T2D and a fasting blood glucose equal to or below 12. Furthermore, the participants in medical treatment should have an HbA1c between 6.1% (43 mmol/mol) and 10% (86 mmol/mol), while participants not receiving medical treatment should have an HbA1c between 6.5% (48 mmol/mol) and 10% (86 mmol/mol). Additionally, an age between 30 and 80 years was required. Patients were excluded if they had alterations in their medical diabetes treatment within the preceding three months, significant comorbidities, such as renal, psychological, neurological, and/or cardiovascular diseases, misuse of substances or alcohol, and current pregnancy or planned conception. The study had a triple-blinded, triple-crossover design, as illustrated in Figure 1A. Each participant received all three interventions, e.g., FAE, AE, and placebo twice daily for eight weeks each, with no less than 3 weeks washout in-between. To further reduce carry-over effects in our analysis, we performed a random sequence generation in Excel and randomized the participants to the order of the interventions using blocks of the six possible orders, which is presented in Figure 1B. Consumption of aronia products aside from the supplements was prohibited from three weeks before the trial and during the trial.

Pre and post each supplementation period, we assessed 24 h ambulatory blood pressure and took fasting blood samples to quantify lipids, adiponectin, and hs-CRP levels.

### 2.3. 24 h Ambulatory Blood Pressure Monitoring

The monitoring of 24 h ambulatory blood pressure was conducted using ambulatory blood pressure monitors (Spacelabs Medical, Ebeltoft, Denmark). The monitors were calibrated to measure blood pressure with a half-hour span between 7:00 AM and 11:00 PM, and with an hour span between 11:00 PM and 7:00 AM Participants recorded wake-up and bed times. Using this information, computer software (Sentinel, Ebeltoft, Denmark, version 11.5.2.13260) calculated systolic and diastolic blood pressure measurements for the entire monitoring period, as well as separately for the sleeping and waking periods. One participant who completed the trial did not complete the blood pressure monitoring due to experiencing pain during the measurements. 

### 2.4. Blood Analyses

Blood samples were collected following an eight-hour fasting period and promptly underwent centrifugation at 4 °C and 3989 RPM for 10 min. The resulting plasma samples were preserved at −80 °C until the end of the study, when samples from all participants were analysed together. 

#### 2.4.1. Hs-CRP and Adiponectin

Hs-CRP and adiponectin concentrations were measured using non-competitive, time-resolved immunofluorometric assays. The method for the determination of hs-CRP has been described previously by Reinhard et al. [24]. The measurement of adiponectin was performed like that of hs-CRP, but with different antibodies (#MAB10651 and #BAM1065, R&D Systems, Minneapolis, Minnesota, USA). In group F, one post-treatment value was found to be below the lower level of detection both in regard to hs-CRP and adiponectin. Only samples from participants completing the study were analysed. 

#### 2.4.2. Lipids

The Department of Clinical Biochemistry at Aarhus University Hospital, Denmark (DS/EN ISO 15189:2013 approved [25]), measured HDL-cholesterol, total cholesterol, and triglycerides. They also provided LDL-cholesterol concentrations which were calculated using the Friedewald formula which is considered the gold standard for determining LDL-cholesterol levels [26]. In two samples, triglyceride concentrations were >4 mmol/L, in which case the LDL-cholesterol concentration could not be calculated.

### 2.5. Power Calculation

Based on a power calculation which has been described previously [23], we calculated that statistical power could be obtained with 18 participants completing the trial. However, due to a rather large dropout rate early in the study, we chose to recruit 36 participants. 

### 2.6. Statistics

Statistical analysis was performed in collaboration with a medical statistician who provided the code for the fitting of a linear mixed effects model in Rstudio (Rstudio, Package lme4). As fixed effects, we included the treatment order (six possible orders), period (first, middle, last), treatment (FAE, AE, placebo), and time (pre, post), along with the interaction between treatment and time. As random effects, we included record id and record id within period. Standard normality tests of residuals were conducted, including quantile-quantile plots and histograms, along with plots of residuals versus fitted values. We used the Rstudio package emmeans to compute estimated marginal means from the mixed model. We will simply refer to the estimated marginal means as ‘means’ throughout this article. We also compared mean differences (Δ-mean) between the three supplementations and obtained the corresponding *p*-values using the package emmeans. For normally distributed data, we present mean or Δ-mean ± standard error. The data on hs-CRP were not normally distributed; therefore, we compared ratios instead of Δ-means. To clarify, a ratio of 1.10 signifies a 10% increase from pre to post, whereas a value of 0.83 denotes a 17% decrease. 

If values were below the lower level of detection in the previously described assays, we imputed these values as half of the lower limit of detection for the respective assays. We assessed the baseline data for normality, and for normally distributed data, we will report the results as mean ± standard deviation (SD). Non-normally distributed data will be reported as the median along with the interquartile range (IQR).

## 3. Results

### 3.1. Baseline characteristics

**Figure 2:**
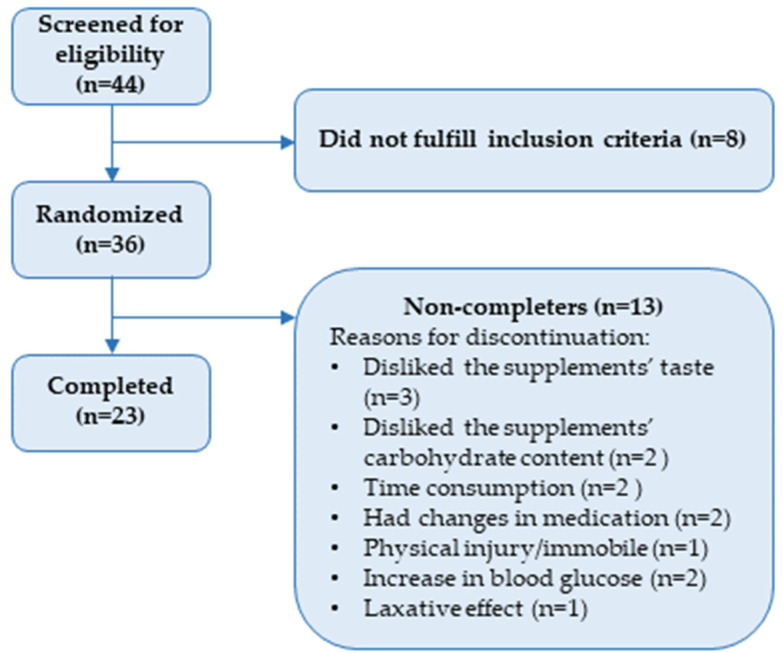
CONSORT diagram with overview of enrolment and discontinuation. Reprinted with permission from [23] under the Creative Commons Attribution (CC BY) license.

As depicted in Figure 2, we assessed 44 participants for eligibility, and we included 36 participants of whom 23 completed the study. We have previously reported the baseline values (see Table 2) [23]. Of the completers of the trial, 15 were men and 8 were women; the mean body mass index was 82.0 ± 16.2 kg/m^2^ and the mean age was 67.6 ± 5.5 years [23]. Of the non-completers, 21 were men and 15 were women; the mean body mass index was 28.6 (24.3–32.0) kg/m^2^ and the mean age was 66.9 ± 6.0 years. The participants continued with their regular medications during the course of the trial (see Table 3).

### 3.2. 24 h Ambulatory Blood Pressure Monitoring

Results were obtained before and after each intervention period. We analysed 24 h measurements, however, no intra- or inter group differences were found (Table 4).

### 3.3. Blood Analyses

Overall, we observed no effects on hs-CRP or adiponectin. However, there was a trend towards improvements upon supplementation with AE, with a 17% decrease in hs-CRP and −0.59 ± 0.26 mg/L decrease in adiponectin. 

**Figure 3:**
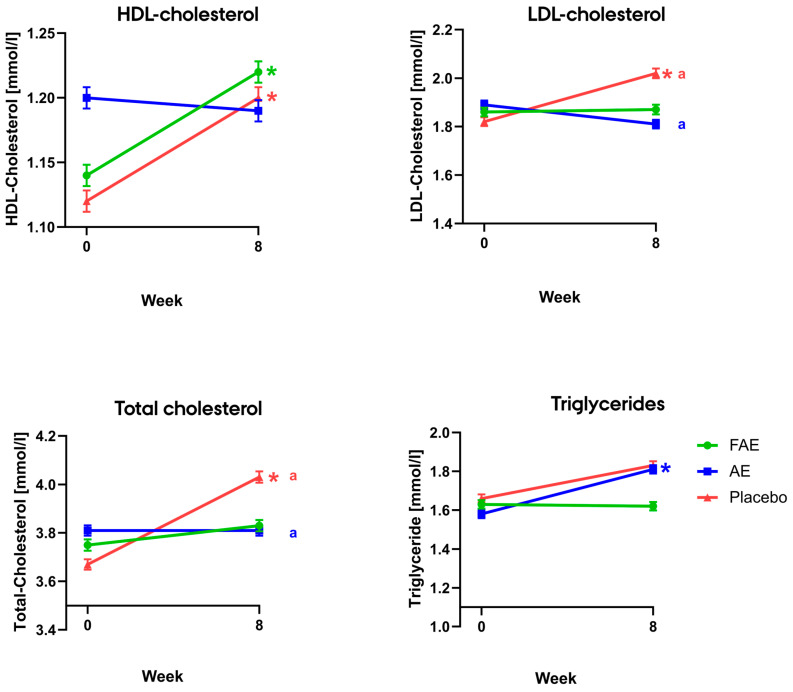
Mean pre and post values for fasting levels of LDL-cholesterol, HDL-cholesterol, total cholesterol, and triglycerides. Data are presented as mean ± standard error. * indicates a significant difference between pre and post values. ^a^ indicates significant differences between Δ-means. Abbreviations: FAE: fermented aronia extract, AE: aronia extract, HDL-cholesterol: high-density lipoprotein cholesterol, LDL-cholesterol: low-density lipoprotein cholesterol.

Regarding lipids, the intra-group analysis revealed several increases distributed across all groups after supplementation, and an overview is provided in Figure 3. Firstly, HDL-cholesterol was increased from 1.14 ± 0.05 to 1.22 ± 0.05 mmol/L (*p* = 0.03) after supplementation with FAE. Secondly, triglycerides were increased from 1.58 ± 0.13 to 1.81 ± 0.13 mmol/L (*p* = 0.03) after supplementation with AE. Lastly, HDL-cholesterol was increased from 1.12 ± 0.05 to 1.20 ± 0.05 mmol/L (*p* = 0.02), LDL-cholesterol was increased from 1.82 ± 0.11 to 2.02 ± 0.12 mmol/L (*p* = 0.01), and total cholesterol was increased from 3.67 ± 0.13 to 4.03 ± 0.14 mmol/L (*p* = 0.0003) in the placebo group. Regarding inter-group differences, ΔLDL-cholesterol and Δtotal-cholesterol were significantly higher in the placebo group compared to the AE group. Specifically, ΔLDL-cholesterol was −0.09 ± 0.07 mmol/L in the AE group and 0.20 ± 0.08 mmol/L in the placebo group (*p* = 0.04), and Δtotal-cholesterol was 0.002 ± 0.08 mmol/L in the AE group versus 0.36 ± 0.09 mmol/L in the placebo group (*p* = 0.01). However, it should be noted that differences within groups were small with changes of maximum 0.1 mmol/L after supplementation with AE and FAE, except for triglycerides, which increased by 0.23 ± 0.08 mmol/L in the AE group. Increases were higher after supplementation with placebo, with increases in triglyceride and LDL-cholesterol of 0.2 mmol/L and in total cholesterol of 0.4 mmol/L. 

## 4. Discussion

In this study, we investigated the effects on cardiovascular risk factors of an eight-week supplementation with aronia extract, fermented aronia extract, and placebo, respectively, in subjects with T2D. After an 8-week supplementation with aronia, we did not observe any significant differences in blood pressure, adiponectin, or hs-CRP. 

Regarding blood pressure, our results are in line with findings from Simeonov et al. who did not find any significant effect on blood pressure in individuals with T2D after a 3-month non-blinded, non-controlled supplementation regimen [27]. Nevertheless, there was a non-significant reduction in systolic blood pressure from 144 to 134 mmHg and in diastolic blood pressure from 91 to 84 mmHg [27]. However, in line with our results, Milutinovic et al. also observed nearly unchanged blood pressure after a three-month supplementation with aronia juice [28]. It should be noted that their study was non-blinded and non-controlled, and they only provided 259 mg anthocyanins per day [28]. Furthermore, in a previous meta-analysis, we assessed the effect of aronia on individuals with increased cardiovascular risk, and we found no effect of aronia either [5]. 

No changes were observed for hs-CRP. However, baseline values were generally low with values below 1 mg/L, and a value below 10 mg/L indicates a low cardiovascular risk [29]. Thus, the baseline hs-CRP values may be too low for us to induce any improvements. In line with our results, Milutinovic et al. found no effect of aronia supplementation on hs-CRP in individuals with T2D either [28]. As far as we are aware, there have been no studies examining the impact of aronia on adiponectin in individuals with T2D. However, the effect has been studied in obese individuals, who had a mean baseline adiponectin level of 7.98 mg/L, which is comparable to our study participants’ values [30]. The researchers observed a significant increase in adiponectin after a two-month supplementation with aronia, which is in contrast to our results. However, the study was non-blinded, and the study population was small (*n* = 10) [30]. Consequently, more studies are needed to verify the findings. 

In accordance with the European Society of Cardiology, it is recommended that individuals with T2D and high CVD risk are more closely monitored in regard to lipid levels than healthy people with LDL-cholesterol levels below 1.8 mmol/L [31]. We observed baseline values below or very close to the recommended ones in all intervention groups, which indicates that the participants were well regulated at baseline. However, there was a significant increase of 0.20 ± 0.08 mmol/L in LDL-cholesterol from 1.82 ± 0.11 to 2.02 ± 0.12 mmol/L (*p =* 0.01) after supplementation with placebo. This increase was accompanied by an increase in total cholesterol from 3.67 ± 0.13 to 4.03 ± 0.14 mmol/L (*p =* 0.0003). For both LDL- and total cholesterol, the Δ-mean in the placebo group was significantly higher than that in the AE group, where the levels were nearly unchanged.

In accordance with to the European Society of Cardiology, it is recommended that fasting triglyceride levels are maintained below 2.3 mmol/L [31]. Thus, the participants had well-regulated levels at baseline. Unfortunately, we observed an unexpected increase in triglycerides of 0.23 ± 0.08 from 1.58 ± 0.13 to 1.81 ± 0.13 (*p* = 0.03) after supplementation with FAE. Likewise, there was also a non-significant increase of 0.17 ± 0.09 mmol/L in triglycerides from 1.66 ± 0.13 to 1.83 ± 0.13 mmol/L after supplementation with placebo. Nevertheless, the levels remained within the recommended range, and the increase was regarded as not clinically relevant. Along with these increases, we also observed a 0.08 ± 0.03 mmol/L increase in HDL-cholesterol from 1.66 ± 0.13 to 1.83 ± 0.13 (*p* = 0.03) after supplementation with AE, and a 0.08 ± 0.03 mmol/L increase from 1.12 ± 0.05 to 1.2 ± 0.05 mmol/L after supplementation with placebo. Concerning HDL-cholesterol, the American Diabetes Association recommends to maintain levels above 1.1 mmol/L for men and 1.2 mmol/L for women, and increasing the level is associated with a decreased risk of cardiovascular events [32]. Thus, at baseline, the levels were already close to the recommended levels, especially in the FAE intervention group, where the mean baseline was 1.2 ± 0.05 mmol/L, which was non-significantly higher than baseline for AE and placebo. It is inexplicable why HDL-cholesterol levels increase after supplementation with FAE and placebo, but not after supplementation with AE. However, it might be because the AE group already had a mean HDL-cholesterol concentration of 1.2 mmol/L. Considering our findings on lipid concentrations, it is worth emphasizing that around 65% of the participants were receiving statin treatment during the study, which potentially mitigates the effects and reduces the effect of the supplements. 

Since placebo, FAE, and AE are similar in nutritional composition, but all contain rather large amounts of sugar, it is interesting that lipid levels changed less than 0.1 mmol/L after supplementation with FAE and AE, with the exception of triglycerides, which were increased by 0.23 ± 0.08 mmol/L after AE supplementation. This could suggest that FAE and AE might have protective effects by preventing rises in lipid levels after the consumption of an unhealthy amount of sugar for eight weeks. On the other hand, it remains puzzling why triglycerides would increase after supplementation with AE. However, a higher intake of saturated fatty acids and a lower intake of polyunsaturated fatty acids and carbohydrates have been connected to lower triglyceride levels [33]. Thus, even though the nutritional composition is very similar in the test products, the slightly different composition of AE in comparison to especially placebo might account for the increase. In summary, AE provides a modestly higher amount of carbohydrates and saturated fatty acids as well as a lower amount of polyunsaturated fatty acids in comparison to placebo. Likewise, AE also provides more carbohydrates than FAE; however, they have the same concentration of polyunsaturated fatty acids. Nevertheless, the fermentation process may have changed the composition of macronutrients and polyphenols into different metabolites with different biological activity. In addition, it is challenging to apply proper blinding in diet-intervention trials, unless capsules are used. Thus, the increase in blood lipid levels in the placebo group could reflect insufficient blinding and be a result of the placebo effect, despite our efforts to make the test products similar in taste, colour, and texture.

Furthermore, it has previously been reported that raisins have a low glycaemic index, and that 50 g of raisins can be considered a healthy snack for individuals with T2D [34,35]. However, we do not know the glycaemic index of our supplements, and an analysis of each test product’s glycaemic index could give important information. It cannot be ruled out that the glycaemic index of our test products is higher than expected, and diets containing food elements with a high glycaemic index may have unfavourable effects on blood lipids with decreased HDL-cholesterol and increased triglyceride levels [36]. In addition, we used a small amount of coconut oil in our test products, i.e., 3 g per daily dose. Coconut oil is solid at room temperature, and therefore important in regarding the test products’ texture. Unfortunately, coconut oil contains 86% saturated fat, and therefore it accounts for the majority of the saturated fats in the test products. The relationship between blood lipids and coconut oil consumption has been examined in other studies that demonstrated a negative impact on LDL-cholesterol, while the results on triglyceride, HDL-, and total cholesterol levels were conflicting [37,38]. Thus, the coconut oil in the snack bars could potentially have contributed to the observed rise in LDL-cholesterol. However, most studies that report a negative impact of coconut oil have used 30–80 g of coconut oil per day as intervention [39,40,41,42]. Importantly, two studies have examined the effect of 5–6 g per day, and they found no negative effects on lipid levels [43,44]. However, we cannot exclude that raisins or coconut oil may have increased the participants’ cholesterol levels. 

Even though the supplements have small variations in nutritional composition, it is unlikely that this alone could account for the observed differences. It is possible that the high content of anthocyanins, in AE and FAE, prevented increases in blood lipids like those observed after supplementation with placebo. In line with this postulation, a meta-analysis has reported that a supplementation with anthocyanins had a positive effect in individuals with lipidaemia [45,46]. If we assume that no change in blood lipids reflects a lipid lowering effect of aronia, our results are partly corroborated by Simeonov et al. and Milutinuvic et al., who found improvements in total cholesterol and LDL-cholesterol, respectively, after aronia supplementation in subjects with T2D [27,28]. In opposition to our findings, Simeonov et al. found an improvement in triglyceride concentration [27], and Milutinovic et al. did not observe any effect on HDL-cholesterol, total cholesterol, or triglycerides [28]. Furthermore, our previously published meta-analysis on the effect of aronia on subjects at increased cardiometabolic risk did not report any effects of aronia on lipid levels [5]. In contrast, another meta-analysis examined the effect of aronia without any requirements regarding the health status of the subjects, and found increases in HDL-cholesterol and decreases in total cholesterol and LDL-cholesterol after the administration of aronia [7]. In addition to the articles included in the meta-analyses, a recently published RCT has demonstrated that a 3-month aronia supplementation has no overall effect on blood cholesterol levels in men, with modest increases in cholesterol levels [47]. However, a subgroup analysis demonstrated improved LDL-cholesterol levels in men below 40 years of age [47]. Thus, there are indications that aronia could have beneficial effects on lipid levels, but as evidence is conflicting, more studies are warranted to draw any conclusion. 

## 5. Conclusions

In conclusion, aronia extracts, both fermented and non-fermented, could contribute to improvements in lipid profiles in subjects with T2D, but more high-quality studies are needed to confirm our findings. Preferably, these studies should be performed using aronia in its pure form to avoid the possible interference of raisins and coconut oil. If such studies could confirm aronia’s presumed lipid-lowering effects, aronia could have a wide application as a supplement not only in relation to T2D but also in relation to cardiometabolic diseases in general. Apparently, aronia does not affect blood pressure, adiponectin, or hs-CRP.

## Figures and Tables

**Table 1:** Nutritional composition of FAE, AE, and placebo. Data are provided for 2 bars, which correspond to each participant’s daily intake. Energy percentages in relation to the total energy content in the bars are stated in square brackets for total fats (9 kcal per gram), total carbohydrates (4 kcal per gram), and protein (4 kcal per gram). Reprinted with permission from [23] under the Creative Commons Attribution (CC BY) license.

Nutritional Content per Daily Dose	FAE	AE	Placebo
Energy [kcal]	234.6	240.1	227.3
Total fats [g] (%)	3.7 (14.2)	4.0 (15.0)	3.5 (13.9)
- Saturated [g]	2.9	3.0	2.4
- Unsaturated [g]	0.4	0.6	0.5
- Polyunsaturated [g]	0.2	0.2	0.5
Total carbohydrates [g] (%)	43.2 (73.7)	45.1 (75.1)	41.1 (72.3)
- Free sugars [g]	38.6	38.6	34.7
Dietary fibres [g]	9.2	6.6	8.1
Protein [g] (%)	2.5 (4.3)	2.5 (4.2)	3.7 (6.5)

Abbreviations: FAE: fermented aronia extract, AE: aronia extract.

**Table 2:** Mean measurements at baseline for completers and non-completers. Reprinted with modifications from [23] under the Creative Commons Attribution (CC BY) license.

Variable (Unit)	Completers (*n* = 23). Value in Mean ± SD or Median (IQR)	Randomized (*n* = 36). Value in Mean ± SD or Median (IQR)
Gender	15 (M) 8 (W)	21 (M) 15 (W)
Age (years)	67.6 ± 5.5	66.9 ± 6.0
Bodyweight (kg)	82.0 ± 16.2	85.9 (72.6–95.5)
Body mass index (kg/m^2^)	26.7 (23.2–29.8)	28.6 (24.3–32.0)
Haemoglobin A1c (mmol/mol)	50.0 (47.5–54)	50.5 (47.0–55.0)

Abbreviations: SD: standard deviation, IQR: interquartile range, W: women, M: men.

**Table 3:** Overview of participants’ medications. Reprinted with modifications from [23] under the Creative Commons Attribution (CC BY) license.

Medication/Compound	Completers (*n* = 23). Received by [Number Participants (%)]	Randomized (*n* = 36). Received by [Number Participants (%)]
Metformin	21 (91.3)	33 (91.7)
Insulin	1 (4.3)	3 (8.3)
GLP-1 receptor agonist	4 (17.4)	6 (16.7)
Dipeptidyl peptidase-4 inhibitor	2 (8.7)	2 (5.6)
Sodium-glucose Cotransporter-2 inhibitor	8 (34.8)	9 (25.0)
Sulfonylurea	2 (8.7)	3 (8.3)
Statins	15 (65.2)	25 (69.4)
ACE inhibitor	10 (43.5)	15 (41.7)
Angiotensin II receptor blocker	4 (17.4)	9 (25.0)
Beta blocker	8 (34.8)	11 (30.6)
Antiplatelet/anticoagulant treatment	6 (26.1)	9 (25.0)
Calcium channel blocker	6 (26.1)	10 (27.8)
Cardiac glycosides	0 (0.0)	1 (2.8)
Diuretics	5 (21.7)	8 (22.2)
Levothyroxine treatment	2 (8.7)	2 (5.6)
Dietary supplement	12 (52.2)	20 (55.6)

**Table 4:** Results for 24 h ambulatory blood pressure monitoring, inflammatory markers, and blood lipid levels. Results are provided as mean difference (Δ-mean) along with pre and post measurements. However, a ratio (confidence level) is used instead of Δ-mean in case of a non-normal distribution. The data on hs-CRP were not normally distributed; therefore, we compared ratios instead of Δ-means. To clarify, a ratio of 1.10 signifies a 10% increase from pre to post, whereas a value of 0.83 denotes a 17% decrease. All pre and post measurements are provided on the scale outlined in the left column ± standard error. Significant differences are given in bold and marked by *. If several significant values are present in one line, differences are marked by superscript letters.

Variable (Unit)		Δ-Mean for FAE		Δ-Mean for AE		Δ-Mean for Placebo		*p* Value
		Pre	Post	Pre	Post	Pre	Post	
24 h systolic BP (mmHg) *n* = 35		−0.71 ± 1.18		0.03 ± 1.07		−0.30 ± 1.17		
		125 ± 2.14	125 ± 2.19	124 ± 2.14	124 ± 2.14	124 ± 2.12	124 ± 2.14	
24 h diastolic BP (mmHg) *n* = 35		0.16 ± 0.77		0.03 ± 0.70		−1.40 ± 0.77		
		71 ± 1.47	71 ± 1.49	71 ± 1.43	71 ± 1.46	71 ± 1.46	71 ± 1.49	
Hs-CRP *n* = 23	(ratio)	1.10 (0.69–1.76)		0.83 (0.52–1.34)		1.05 (0.66–1.68)		
	(mg/L)	0.56 ± 0.11	0.62 ± 0.12	0.69 ± 0.14	0.58 ± 0.12	0.53 ± 0.10	0.55 ± 0.11	
Adiponectin (mg/L) *n* = 23		0.14 ± 0.26		−0.59 ± 0.26		0.06 ± 0.26		
		8.44 ± 0.87	8.58 ± 0.87	9.14 ± 0.87	8.55 ± 0.87	8.38 ± 0.87	8.43 ± 0.87	
HDL-cholesterol (mmol/L) *n* = 36		0.08 ± 0.03		−0.01 ± 0.03		0.08 ± 0.03		
		**1.14 ± 0.05 ^a^**	**1.22 ± 0.05 ^a^**	1.2 ± 0.05	1.19 ± 0.05	**1.12 ± 0.05 ^b^**	**1.2 ± 0.05 ^b^**	^a^ 0.03 ^b^ 0.02
LDL-cholesterol (mmol/L) *n* = 36		0.01 ± 0.08		−0.09 ± 0.07 *		0.20 ± 0.08 *		0.04
		1.86 ± 0.11	1.87 ± 0.12	1.89 ± 0.11	1.81 ± 0.11	1.82 ± 0.11 *	2.02 ± 0.12 *	0.01
Total-cholesterol (mmol/L) *n* = 36		0.08 ± 0.09		0.002 ± 0.08 *		0.36 ± 0.09 *		0.01
		3.75 ± 0.14	3.83 ± 0.14	3.81 ± 0.13	3.81 ± 0.13	3.67 ± 0.13 *	4.03 ± 0.14 *	0.0003
Triglyceride (mmol/L) *n* = 36		−0.01 ± 0.09		0.23 ± 0.08		0.17 ± 0.09		
		1.63 ± 0.13	1.62 ± 0.13	1.58 ± 0.13 *	1.81 ± 0.13 *	1.66 ± 0.13	1.83 ± 0.13	0.03

Abbreviations: FAE: fermented aronia extract, AE: aronia extract, BP: blood pressure, Hs-CRP: high-sensitive C-reactive protein, HDL-cholesterol: high-density lipoprotein cholesterol, LDL-cholesterol: low-density lipoprotein cholesterol.

## Data Availability

Data supporting reported results can be obtained by contacting the corresponding author.
